# Influence of pharmaceutical marketing on Medicare prescriptions in the District of Columbia

**DOI:** 10.1371/journal.pone.0186060

**Published:** 2017-10-25

**Authors:** Susan F. Wood, Joanna Podrasky, Meghan A. McMonagle, Janani Raveendran, Tyler Bysshe, Alycia Hogenmiller, Adriane Fugh-Berman

**Affiliations:** 1 Department of Health Policy and Management, George Washington University Milken Institute School of Public Health, Washington DC, United States of America; 2 Department of Infection Prevention and Control, JPS Health Network, Fort Worth, Texas, United States of America; 3 Department of Regional Planning, MedStar Health, Columbia, Maryland, United States of America; 4 George Washington University School of Medicine, Washington DC, United States of America; 5 National Opinion Research Center at the University of Chicago, Bethesda, Maryland, United States of America; 6 Department of Pharmacology and Physiology, Georgetown University Medical Center, Washington DC, United States of America; Jagiellonian University, POLAND

## Abstract

**Importance:**

Gifts from pharmaceutical companies are believed to influence prescribing behavior, but few studies have addressed the association between industry gifts to physicians and drug costs, prescription volume, or preference for generic drugs. Even less research addresses the effect of gifts on the prescribing behavior of nurse practitioners (NPs), physician assistants (PAs), and podiatrists.

**Objective:**

To analyze the association between gifts provided by pharmaceutical companies to individual prescribers in Washington DC and the number of prescriptions, cost of prescriptions, and proportion of branded prescriptions for each prescriber.

**Design:**

Gifts data from the District of Columbia’s (DC) AccessRx program and the federal Center for Medicare and Medicaid Services (CMS) Open Payments program were analyzed with claims data from the CMS 2013 Medicare Provider Utilization and Payment Data.

**Setting:**

Washington DC, 2013

**Participants:**

Physicians, nurse practitioners, physician assistants, podiatrists, and other licensed Medicare Part D prescribers who participated in Medicare Part D (a Federal prescription drug program that covers patients over age 65 or who are disabled).

**Exposure(s):**

Gifts to healthcare prescribers (including cash, meals, and ownership interests) from pharmaceutical companies.

**Main outcomes and measures:**

Average number of Medicare Part D claims per prescriber, number of claims per patient, cost per claim, and proportion of branded claims.

**Results:**

In 2013, 1,122 (39.1%) of 2,873 Medicare Part D prescribers received gifts from pharmaceutical companies totaling $3.9 million in 2013. Compared to non-gift recipients, gift recipients prescribed 2.3 more claims per patient, prescribed medications costing $50 more per claim, and prescribed 7.8% more branded drugs. In six specialties (General Internal Medicine, Family Medicine, Obstetrics/Gynecology, Urology, Ophthalmology, and Dermatology), gifts were associated with a significantly increased average cost of claims. For Internal Medicine, Family Medicine, and Ophthalmology, gifts were associated with more branded claims. Gift acceptance was associated with increased average cost per claim for PAs and NPs. Gift acceptance was also associated with higher proportion of branded claims for PAs but not NPs. Physicians who received small gifts (less than $500 annually) had more expensive claims ($114 vs. $85) and more branded claims (30.3% vs. 25.7%) than physicians who received no gifts. Those receiving large gifts (greater than $500 annually) had the highest average costs per claim ($189) and branded claims (39.9%) than other groups. All differences were statistically significant (p<0.05).

**Conclusions and relevance:**

Gifts from pharmaceutical companies are associated with more prescriptions per patient, more costly prescriptions, and a higher proportion of branded prescriptions with variation across specialties. Gifts of any size had an effect and larger gifts elicited a larger impact on prescribing behaviors. Our study confirms and expands on previous work showing that industry gifts are associated with more expensive prescriptions and more branded prescriptions. Industry gifts influence prescribing behavior, may have adverse public health implications, and should be banned.

## Background

Pharmaceutical companies influence healthcare providers’ attitudes and therapeutic choices through financial incentives that include research grants, educational grants, consulting fees, speaker fees, gifts, and meals. Although pharmaceutical company promotion influences a physician’s prescribing behavior,[1–5] studies have consistently shown that physicians do not believe that promotion affects their own prescribing.[6–12]

Less information is available on the influence of industry on physician assistants (PAs), nurse practitioners (NPs), optometrists, podiatrists, and other healthcare providers who prescribe medications. The National Ambulatory Medical Care Survey (NAMCS), a national survey that assesses the use of medical services in the United States,[13] found that NPs and PAs write just as many prescriptions as physicians.[14] The number of prescriptions written by NPs and PAs has more than doubled over the past five years; in 2015, NPs and PAs wrote 676 million of 4.4 billion (15.4%) prescriptions in the U.S.[15] Only a few studies have documented the influence of pharmaceutical promotions and marketing activities on the prescribing of advanced practice nurses.[16–18] No studies were identified on the influence of industry on PAs.

Gifts, no matter their size, have a powerful effect on human relationships. Reciprocity is a strong guiding principle of human interaction.[19–21] Even gifts of small value, such as “modest” industry-sponsored lunches, may foster a subconscious obligation to reciprocate through changes in prescribing practices.[20] DeJong et al has shown that a meal with a value of less than $20 can increase the prescribing of branded statins, beta-blockers, ACE inhibitors, and antidepressants.[22]

Recently available public data show that industry gifts are common among physicians in general [23] and among specialists (i.e. surgeons,[24] emergency medicine physicians,[25] obstetricians/gynecologists,[26] radiation oncologists,[27] gynecologic oncologists,[28] otolaryngologists,[29] and pediatricians.[30]) One study has shown that industry gifts are associated with more expensive prescriptions for Medicare patients (in the U.S., Medicare is Federally-funded insurance that covers patients over 65 and disabled individuals).[4] Another study has found that industry gifts to physicians in Massachusetts increased prescribing of branded statins.[31]

Nationally, no laws prohibit the acceptance of gifts or payments from industry by healthcare providers. Several states restrict gift acceptance; Minnesota, for example, limits gifts and food to $50 per year and limits payments for speaking to “reasonable” fees for “bona fide” education.[32] In Washington DC, members of a medication advisory committee are not allowed to accept a gift from a pharmaceutical company.[33]

In 2009, the Pharmaceutical Research and Manufacturers of America (PhRMA), the trade group for pharmaceutical companies, instituted a voluntary gift restriction policy, recommending that companies not provide any entertainment or recreational items, “reminder” items (i.e. pens, notepads, mugs), or gifts intended for entertainment or recreation. Gifts intended for education or patient care are allowed if they do not cost more than $100.[34]

The 2004 AccessRx Act requires pharmaceutical companies to report all pharmaceutical marketing expenditures, including gifts to all persons and entities licensed to provide healthcare in the District (i.e. physicians, nurses, physician assistants, and pharmacists), salaries spent on pharmaceutical representatives and other pharmaceutical marketers, and advertising. A portion of this law was pre-empted when the federal Centers for Medicare and Medicaid Service (CMS) Open Payments system went into effect in August 2013. Open Payments collects information on gifts to doctors (i.e. Doctor of Medicine, Doctor of Osteopathy, Doctor of Dentistry, Doctor of Dental Surgery, Doctor of Podiatry, Doctor of Optometry, Doctor of Chiropractic Medicine).

The release of the CMS Medicare Provider Utilization and Payment Data, which summarizes the utilization and payments for procedures, services, and prescription drugs provided by organizational or individual providers to Medicare patients,[35] provided a unique opportunity to examine prescribing behavior. Combining CMS Part D Prescriber data (Medicare Part D is prescription coverage) with data from Washington DC Department of Health’s (DOH) AccessRx and CMS Open Payments allowed us to gain insight into the impact of pharmaceutical marketing efforts on individual prescribing behavior.

## Methods

### Study design

Primary outcomes of this study included the average number of Medicare claims per prescriber, claims per patient, cost per claim, and proportion of branded claims. A claim was classified as branded or generic within the CMS Medicare Part D data set. The average number of claims per prescriber was calculated by dividing the total number of claims for all prescribers by the total number of prescribers included in the study. The average number of claims per patient was calculated by dividing the total number of claims by the total number of Medicare Part D patients associated with each prescriber. The average cost per claim was calculated by dividing the total cost of claims by the total number of claims. The proportion of branded claims was calculated by dividing the total number of claims of branded drugs by the total number of claims. This study was approved via expedited review by the George Washington University’s Institutional Review Board under IRB #060942.

### Data sources

This analysis combined pharmaceutical marketing data from the DC DOH AccessRx program and the CMS Open Payments program with Medicare Part D claims data from CMS’ Medicare Provider Utilization and Payment Data. Our study included all gifts provided in 2013. Physician gifts from January to July 2013 were obtained from AccessRx. Physician gifts from August to December 2013 were obtained from Open Payments. The reason for switching databases was that reporting of physician gifts to Open Payments began in August 2013, which pre-empted reporting of physician gifts to AccessRx. Information for NPs, PAs, and other non-physician prescribers were obtained entirely from the AccessRx database, as Open Payments does not gather these data. Open Payments, but not AccessRx, collects data on payments from medical device companies. For consistency, we identified all manufacturers of devices and medical supplies through online searches and excluded these from analyses, thus limiting our analysis to pharmaceutical companies.

There are minor differences in reporting requirements between AccessRx and Open Payments. AccessRx requires reporting of expenses more than $25 made by a manufacturer or labeler (repackager) of prescription drugs associated with educational or informational programs, food, entertainment, gifts, travel, and product samples.[36] Information excluded from reporting includes “reasonable compensation” in connection with a clinical trial, scholarships for certain conferences, and samples that will be distributed free of charge to patients. Gifts reportable to Open Payments in 2013 include payments of $10 or more from any manufacturers of drugs, devices, biologics, or medical supplies covered by Medicare, Medicaid, or the Children’s Health Insurance Program to a physician or teaching hospital.[37] Information excluded from reporting includes manufacturers who make less than 10% gross revenue from covered products and drug samples for patients.

### Participants

Medicare Part D prescribers in DC, including physicians, nurse practitioners, and physician assistants, were identified by inclusion in the 2013 CMS Medicare Provider Utilization and Payment Data.

Gift recipients were identified based on inclusion in either the AccessRx or Open Payments database. Prescribers who submitted more than ten Medicare claims in 2013 and appeared in either AccessRx or Open Payments as receiving a gift of any value were recorded as gift recipients (CMS excludes claim counts of ten or fewer from the Medicare Provider Utilization and Payment database). Prescribers who submitted Medicare claims and appeared in the database, but whose names did not appear in either the AccessRx or Open Payments datasets, were assumed to be non-gift recipients.

Names were used to match Medicare claims in CMS’ Medicare Provider Utilization and Payment Data to gifts reported in AccessRx and Open Payments. Although the U.S. used national provider identifiers (NPIs), a unique 10-digit number for each healthcare provider, we did not use NPI numbers because they were not available in all databases. Instead, we used middle initials, facility affiliations, and specialty information to assist with matching records. When there were inconsistencies in the spelling of names and other identifiers between databases, a manual search for a gift recipient’s name was performed in other fields and efforts were made to identify prescribers by address or affiliation. Healthcare prescribers were excluded from the analysis only if all of these tactics failed.

Specialties were determined through CMS Medicare Provider Utilization and Payment data and by recipient credentials included in the AccessRx data. Internal Medicine subspecialties (Cardiology, Endocrinology, Gastroenterology, Infectious Disease, Pulmonary Disease), Family Medicine, Obstetrics/Gynecology, Urology, Opthalmology, Dermatology, Emergency Medicine, Psychiatry and its subspecialties (General Psychiatry, Psychiatry/Neurology, Neuropsychiatry), and Other Surgery Specialties (Cardiothoracic Surgery, Colorectal Surgery, Oral and Maxillofacial Surgery, Plastic and Reconstructive Surgery, Vascular Surgery, Neurosurgery, Thoracic and Vascular Surgery) were grouped into categories (Table 1). Subspecialties with small numbers were combined in order to increase the statistical power in the analysis. Specialties with fewer than five individuals were excluded to avoid the bias associated with small sample sizes.

**Table 1:** Included specialties and subspecialties.

Internal Medicine
Internal Medicine Subspecialties included:
• Cardiology
• Endocrinology
• Gastroenterology
• Infectious Disease
• Pulmonary Disease
Family Medicine
Pediatric Medicine
Ophthalmology
Dermatology
Nephrology
Emergency Medicine
Neurology
Psychiatric Subspecialties included:
• Psychiatry
• Geriatric Psychiatry
• Psychiatry/Neurology
• Neuropsychiatry
Radiation Oncology
Diagnostic Radiology
Obstetrics/Gynecology
Urology
General Surgery
Orthopedic Surgery
Other Surgical Specialties included:
• Cardiothoracic Surgery
• Colorectal Surgery
• Oral and Maxillofacial Surgery
• Plastic and Reconstructive Surgery
• Vascular Surgery
• Neurosurgery
• Thoracic and Vascular Surgery
Nurse Practitioners
Physician Assistants
Podiatrists

### Data analysis

The impact of gifts on prescribing patterns was analyzed by comparing gift recipients (n = 1,123) to non-gift recipients (n = 1,750). Subset analyses examined differences by specialty and gift value. The mean values of the average cost per claim and proportion of branded claims were tested using a one-way analysis of variance (ANOVA) and Scheffé post-hoc comparisons with the level of significance of p < 0.05.

Subset analyses were performed to determine whether those receiving small gifts (n = 798), defined as gifts totaling less than $500 year affected prescribing patterns compared to non-gift recipients (n = 1,750) and those receiving gifts greater than $500 (n = 324). A two-tailed T-test was used to compare all differences.

## Results

### Analysis of healthcare prescribers as a whole

In 2013, 1,122 (39.1%) of the 2,873 Medicare Part D prescribers in Washington DC included in our analysis received gifts totaling $3.9 million. The remaining 1,750 (60.9%) prescribers were considered non-gift recipients. Gifts ranged from a total of $7 to more than $200,000 per prescriber. Payments of more than $200,000 to one individual were all monetary payments.

Gift recipients prescribed an average of 892 claims each, more than twice as many as the 389 claims per prescriber for non-gift recipients. Gift recipients submitted significantly more claims per patient (8.8 vs. 6.5) and had a greater average cost per claim ($135 vs. $85). On average, one of three prescriptions (33.5%) written by gift recipients was for a branded drug, compared to one of four prescriptions (25.7%) written by non-gift recipients. All results were significant (p<0.0001) (Table 2).

**Table 2:** Average number of claims per prescriber, claims per patient, cost per claim, and proportion of branded drugs. Medicare Prescribers by Gift Status.

Recipient Type	Average Number of Claims Per Prescriber	Average Number of Claims Per Patient	Average Cost Per Claim	Average Proportion of Branded Claims
**Gift Recipients** (n = 1122)	892 (39%)	8.8	$135	33.5%
**Non-Gift Recipients** (n = 1750)	389 (61%)	6.5	$85	25.7%

All values significantly different with a p value <.0001.

### Analysis by specialty

**Fig 1:**
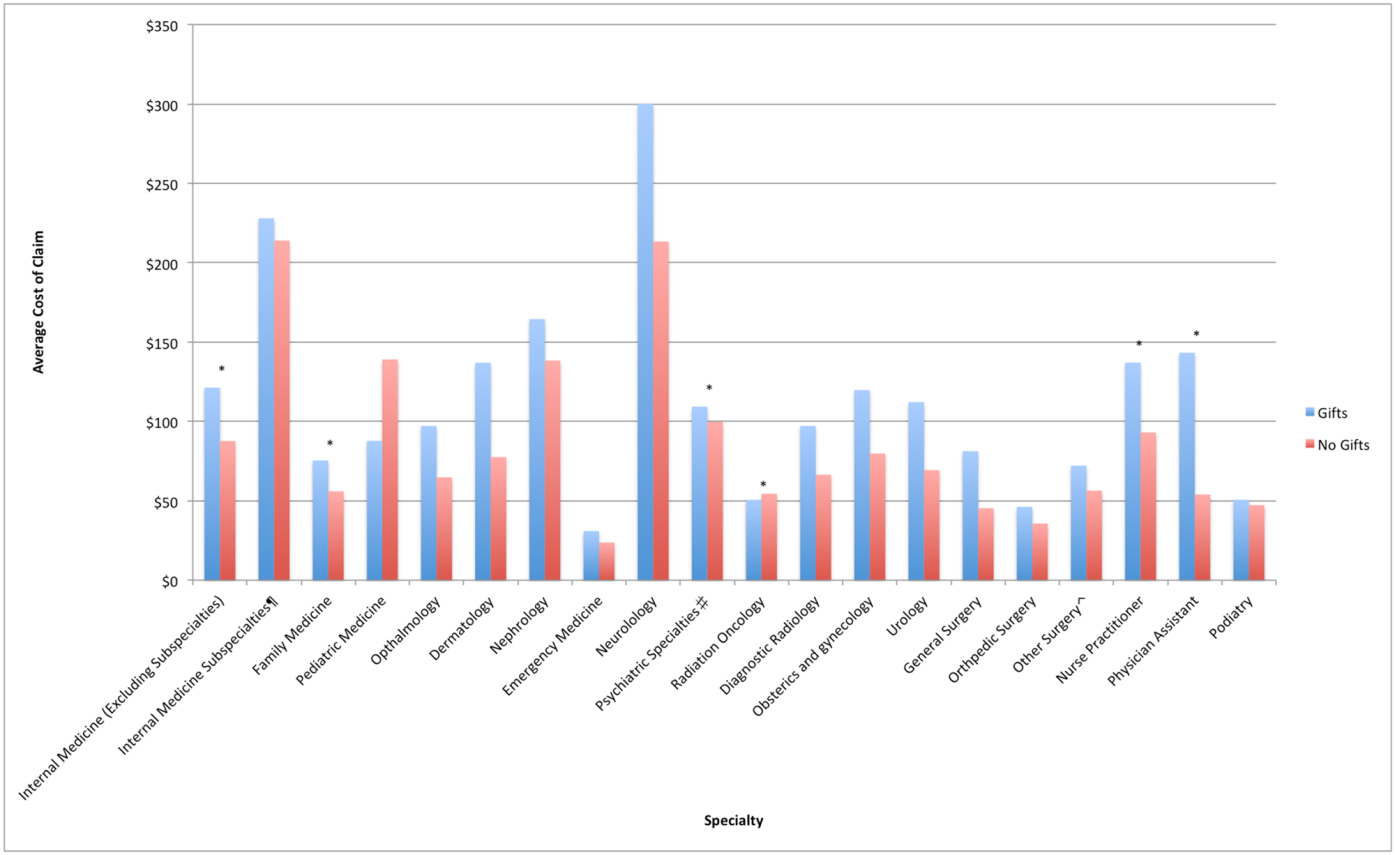
Average cost of claims by specialty for gift and non-gift recipients. Average cost of claims by specialty for gift and non-gift recipients. ¶ Internal Medicine Specialties includes Cardiology, Endocrinology, Gastroenterology, Infectious Disease, Pulmonary Disease. ♯ Psychiatric Specialties includes Psychiatry, Psychiatry & Neurology, Neuropsychiatry, Geriatric Psychiatry. ⌃ Other Surgery includes Cardiac, Colorectal, Maxillofacial, Oral and Maxillofacial, Plastic and Reconstructive, Plastic, Neurological, Thoracic and Vascular Surgery. *Statistically Significant (p<0.05).

**Fig 2:**
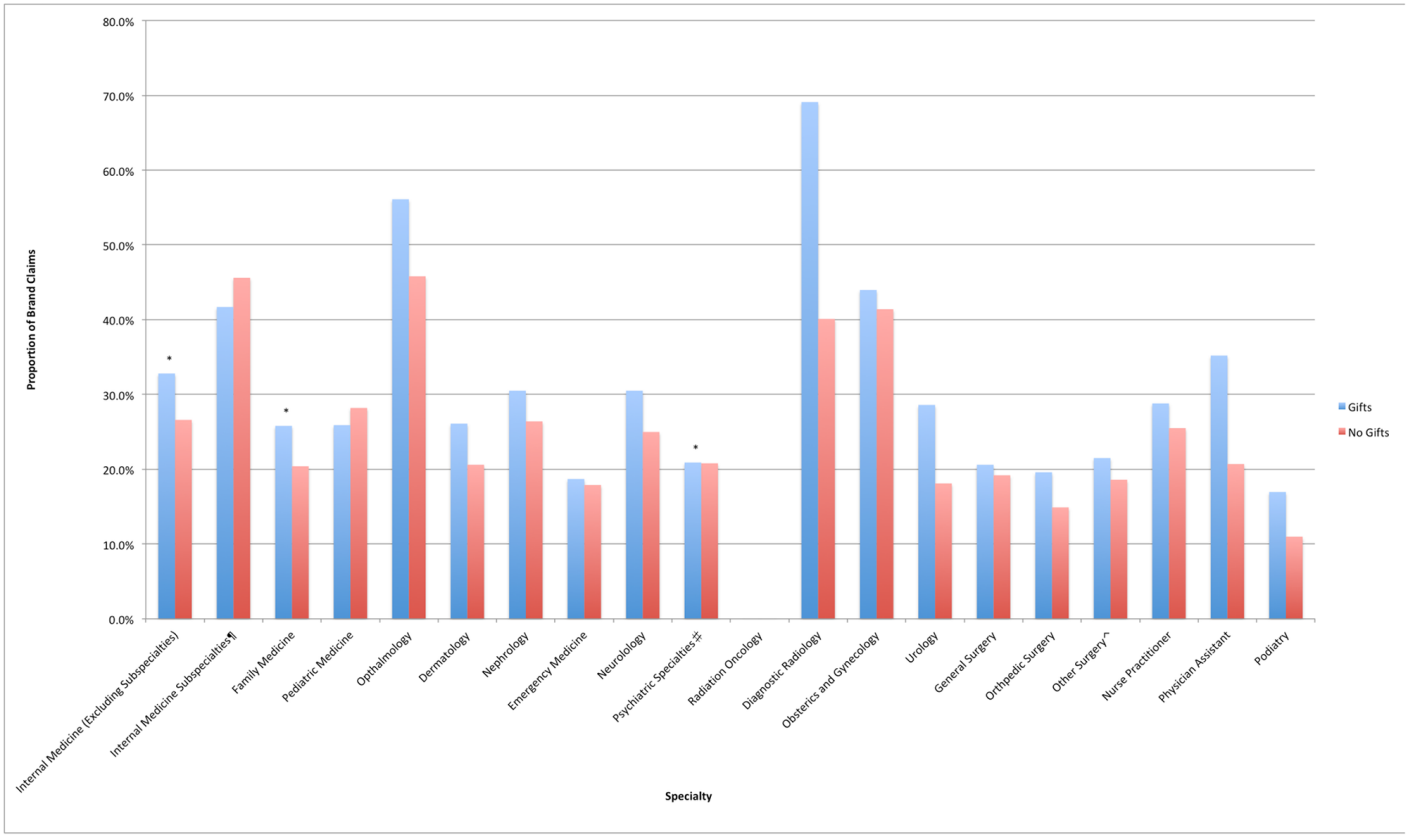
Proportion of branded claims by specialty for gift and non-gift recipients. Proportion of branded claims by specialty by gift and non-gift recipients. ¶ Internal Medicine Specialties includes Cardiology, Endocrinology, Gastroenterology, Infectious Disease, Pulmonary Disease. ♯ Psychiatric Specialties includes Psychiatry, Psychiatry & Neurology, Neuropsychiatry, Geriatric Psychiatry. ⌃ Other Surgery includes Cardiac, Colorectal, Maxillofacial, Oral and Maxillofacial, Plastic and Reconstructive, Plastic, Neurological, Thoracic and Vascular Surgery. *Statistically Significant (p<0.05).

In six specialties (Internal Medicine, Family Medicine, Obstetrics/Gynecology, Urology, Ophthalmology, and Dermatology), gift acceptance was associated with significantly increased average cost per claim (Fig 1). For three of these specialties (Internal Medicine, Family Medicine, and Ophthalmology), gift acceptance was also associated with a higher proportion of branded claims (Fig 2). In eleven other specialties, gift acceptance was not associated with a higher average cost per claim or proportion of branded claims. These included Emergency Medicine, Nephrology, other Internal Medicine subspecialties (Cardiology, Endocrinology, Gastroenterology, Infectious Disease, Pulmonary Disease), Neurology, Pediatrics, Psychiatric subspecialties, Radiation Oncology, Diagnostic Radiology, General Surgery, Orthopedic Surgery, and Other Surgical specialties.

### Physician assistants, nurse practitioners, and podiatrists

Gift acceptance was associated with a significant increase in the average cost per claim for PAs ($213 vs. $63) and NPs ($180 vs. $86). Gift acceptance by PAs was also associated with a significantly higher proportion of branded claims (30.2% vs. 17.0%); NPs who accepted gifts did not have a significantly higher proportion of branded claims (18.3% vs. 16.9%). Gift acceptance by podiatrists was not associated with a higher average cost per claim or proportion of branded claims.

### Analysis by gift value

Acceptance of even small gifts (less than $500 per year) was associated with prescribing outcomes, with larger gifts (greater than $500 per year) having a greater effect on prescribing patterns. The 798 prescribers who received small gifts had a significantly larger average cost per claim in comparison to non-gift recipients ($114 vs. $85). Small-gift recipients also had a significantly higher proportion of branded claims compared to non-gift recipients (30.3% vs. 25.7%). Those prescribers receiving larger gifts had an even larger cost per claim ($189) and larger proportion of branded claims (39.9%) compared to non-gift and small-gift recipients (Table 3).

**Table 3:** Average cost per claim and proportion of branded claims by gift value.

Recipient Type	Average Cost per Claim	Average Proportion of Branded Claims
**Non-Gift Recipients**	$85 (n = 1750)	25.7% (n = 808)
**Small Gift Recipients** ($7-$500)	$114 (n = 798)	30.3% (n = 458)
**Large Gift Recipients** (>$500)	$189 (n = 324)	39.9% (n = 222)

All differences are significant with a p value <.001.

## Discussion

In 2013, Medicare Part D prescribers in DC who received gifts from the pharmaceutical industry generated more prescriptions per prescriber, more prescriptions per patient, more costly prescriptions, and more branded prescriptions. Prescribers who received gifts wrote an average of two more prescriptions per patient, compared to prescribers who did not receive gifts. The discrepancy in number of prescriptions per patient could result from gift recipients treating a sicker population who require more medications, but this is unlikely. It is also possible that prescribers who avoid industry gifts are innately more conservative prescribers. The more likely explanation, however, is that receiving gifts fosters more frequent and more expensive prescriptions.

The direction of causation could be reversed–it is possible that healthcare providers who prescribe more expensive drugs are subsequently targeted for gifts. Even if that were true, the end result remains the same: gifts contribute to maintaining prescribing patterns that are beneficial to industry.

Gifts had a dose-response effect on the average cost per claim and the proportion of branded claims. Even small gifts may have a large impact on prescribing practices. Our results show that healthcare providers who received gifts totaling less than $500 a year prescribed more expensive medications than prescribers who did not receive gifts ($114 vs. $85), with even higher cost and proportion of branded drugs prescribed by those receiving larger gifts.

The effect of gift acceptance by prescribers, especially primary care providers (PCPs), has profound implications for healthcare costs in the U.S. Half (51.3%) of all visits to office-based physicians are to PCPs, who represent about a third of the 624,434 physicians in the U.S. PCPs include family medicine physicians, general practitioners, internists, pediatricians, and geriatricians. Internal medicine (71,487) and family medicine (79,831) are the largest specialties in the U.S., comprising about half of all practicing PCPs.[38] Most PAs and NPs also provide primary care. In this study, PCPs who received gifts–internists, family medicine physicians, NPs, and PAs–had higher average costs per claim and proportion of branded claims. Pediatricians were the only group of PCPs for whom gifts did not appear to be associated with higher average costs per claim and proportion of branded claims.

Healthcare quality may also be affected through pharmaceutical marketing presentations. Gifts of meals and local speaking engagements are usually provided by pharmaceutical representatives, who use gifts, friendship, and flattery to develop relationships with healthcare prescribers that foster receptivity to sales pitches.[21] Although healthcare providers believe they can extract educational information from drug representatives, when tested, prescribers often cannot distinguish between correct and incorrect information.[10, 11] While healthcare providers believe that they accorded scientific materials more weight than biased promotional materials, one study found that their beliefs regarding two common drugs correlated more strongly with promotional materials than with scientific materials.[12]

Restricting industry gifts can affect therapeutic choices. A recent study of fourteen U.S. medical schools that restricted industry gifts found that physicians who had graduated from the schools after the implementation of the gift restriction policies were less likely to prescribe two of three newly introduced psychotropic medications than those who had graduated prior to the gift restrictions.[39] Therefore, it would be beneficial to healthcare systems and practices to restrict interactions between industry representatives and prescribers.

### Strengths and limitations

The strength of this study lies in combining publicly available information from CMS Open Payments with information from DC’s AccessRx program. Each database had only partial information on 2013, but by combining the databases, we could track payments for the full year. While disclosure of payments in the CMS Open Payments database is limited to physicians and teaching hospitals, DC’s AccessRx program is far more comprehensive, tracking pharmaceutical spending on PAs, nurses, APNs, pharmacists, non-teaching hospitals, professional organizations, and universities. In addition, Washington, DC is the only jurisdiction in the U.S. that tracks expenditures on pharmaceutical advertising and salaries for employees of pharmaceutical companies.

One of the limitations of this study is the inability to establish causation. Our study also cannot predict prescribing patterns across different patient demographics. Our analysis is limited to patients enrolled in the Medicare Part D prescription drug program, the vast majority of whom are over age 65. Most clinicians who see patients covered by Medicare Part D would also be seeing patients covered by other insurance programs. It is possible that healthcare providers who see Medicare patients prescribe differently to patients with private insurance, covered under Medicaid (the Federal program for individuals living in poverty), or without insurance. It is also possible that prescribers who do not see Medicare patients prescribe differently than prescribers who see Medicare patients. Both scenarios seem unlikely.

Within the CMS Medicare Provider Utilization and Payment database, prescribers who generate fewer than eleven claims or who have fewer than eleven Medicare patients are excluded. Thus, the data analyzed accounts for 86.8% of claims and 78.1% of total costs in the Medicare Part D program. Comparing gift recipient information resulted in approximately 23.2% of gift recipients being excluded from the analysis of the average number of claims per patient, and 48.2% of gift recipients being excluded from the analysis of the proportion of branded drugs. It is possible that excluded individuals differ from included individuals but this is unlikely. The reporting limits in the programs may result in an underestimation of gifts to prescribers.

We did not assess the health status of patient populations. However, gift recipients were compared to non-gift recipients within specialties, so differences in availability of generic drugs for conditions treated in different specialties should not be a concern.

Despite the study limitations listed above, our data supports the basic premise that industry gifts influence the prescribing patterns of healthcare providers.

Our study aligns with a national report from ProPublica that linked 2014 Medicare Part D data with Open Payments data to examine the effect of gifts on prescribing for 150,323 physicians in five specialties–internal medicine, family medicine, cardiology, psychiatry, and ophthalmology. In all of these specialties, physicians who received any gifts from pharmaceutical or device manufacturers prescribed a higher proportion of branded drugs than physicians who did not receive gifts. Physicians who received industry gifts were two to three times more likely to prescribe branded drugs at very high rates compared to others in the same specialty. ProPublica also analyzed gifts by type of payment and found that physicians who received speaking payments had the highest rates of branded drug prescribing compared to those who received other types of gifts. However, physicians who received a meal increased their branded drug prescribing when compared to physicians who did not receive gifts.[5]

This study also aligns with a national study that examined 725,169 physicians who prescribed medications through Medicare Part D and found that 341,644 physicians (47.1%) were reported as receiving gifts in Open Payments. Being in the top quintile of gift recipients was associated with increased cost per patient and proportion of branded prescriptions.[4]

The association between prescription frequency and cost suggests that industry influence is related to prescription drug costs. This poses both a financial burden on the healthcare system and individual patients. Further, this influence may have adverse public health implications, including polypharmacy and overtreatment that may decrease the quality of care through adverse drug reactions.

## Conclusions

In Washington, DC, healthcare providers who received gifts of any size from pharmaceutical companies generated more prescriptions per patient, more costly prescriptions, and a higher proportion of branded prescriptions compared to healthcare providers who did not receive gifts. Our study confirms and expands upon previous work showing that industry gifts are associated with more expensive prescriptions and more branded prescriptions. The impact of pharmaceutical marketing could have a profound effect on healthcare costs. Because DC is unique in the breadth of data it gathers on non-physician prescribers and costs associated with marketing personnel, this study is more robust than any state could do and demonstrates the impact of marketing on healthcare costs in DC. We recommend further research on the influence of pharmaceutical marketing on patient care. We also recommend further education for healthcare providers about the influence of industry marketing on prescribing behavior. Federal and state agencies are encouraged to maintain and further develop reporting standards for industry marketing activities. Continued research on the influence of industry marketing on prescribing practices, continuous and robust pharmaceutical marketing restrictions, and monitoring and expanding public access to data is needed to mitigate conflicts of interest in healthcare. State and Federal governments should consider restricting pharmaceutical marketing on the grounds that it compromises public health. Industry gifts influence prescribing behavior, may have adverse public health implications, and should be banned.
